# Microplastics and anthropogenic fibre concentrations in lakes reflect surrounding land use

**DOI:** 10.1371/journal.pbio.3001389

**Published:** 2021-09-14

**Authors:** Andrew J. Tanentzap, Samuel Cottingham, Jérémy Fonvielle, Isobel Riley, Lucy M. Walker, Samuel G. Woodman, Danai Kontou, Christian M. Pichler, Erwin Reisner, Laurent Lebreton

**Affiliations:** 1 Ecosystems and Global Change Group, Department of Plant Sciences, University of Cambridge, Cambridge, United Kingdom; 2 Yusuf Hamied Department of Chemistry, University of Cambridge, Cambridge, United Kingdom; 3 The Ocean Cleanup, Rotterdam, the Netherlands; 4 The Modelling House, Raglan, New Zealand; NETHERLANDS

## Abstract

Pollution from microplastics and anthropogenic fibres threatens lakes, but we know little about what factors predict its accumulation. Lakes may be especially contaminated because of long water retention times and proximity to pollution sources. Here, we surveyed anthropogenic microparticles, i.e., microplastics and anthropogenic fibres, in surface waters of 67 European lakes spanning 30° of latitude and large environmental gradients. By collating data from >2,100 published net tows, we found that microparticle concentrations in our field survey were higher than previously reported in lakes and comparable to rivers and oceans. We then related microparticle concentrations in our field survey to surrounding land use, water chemistry, and plastic emissions to sites estimated from local hydrology, population density, and waste production. Microparticle concentrations in European lakes quadrupled as both estimated mismanaged waste inputs and wastewater treatment loads increased in catchments. Concentrations decreased by 2 and 5 times over the range of surrounding forest cover and potential in-lake biodegradation, respectively. As anthropogenic debris continues to pollute the environment, our data will help contextualise future work, and our models can inform control and remediation efforts.

## Abstract 2

Pollution from microplastics and anthropogenic fibres threatens lakes, but we know little about what factors predict its accumulation. This study uses a survey of 67 European lakes spanning 30° of latitude to show that economic and environmental indicators predict the pollution of lakes by anthropogenic microparticles.

## Introduction

Predicting where anthropogenic debris accumulates in aquatic ecosystems is necessary for its control and environmental remediation, but relatively little is known about its distribution outside of the oceans [1,2]. Microparticles that come from synthetic (i.e., plastic) polymers and regenerated or processed natural materials used in textiles are particularly concerning for at least two reasons. First, the small size and wide distribution of microparticles—often defined in surface waters as particles between 300 μm and 5 mm [3]—makes them easily ingested by microscopic to large animals, posing distinct toxic hazards [4–6]. Fibre microparticles are, for example, the most common size class of anthropogenic debris reported to be ingested by marine vertebrates [7]. Microparticles can also support denser biofilm colonies than larger debris and become aggregated with microbial cells [8]. The resulting aggregates can change nitrogen [9] and carbon cycling [10] and even subsidise energy transfer through the food web [11]. Second, microparticles are the eventual endpoint of larger products that fragment and degrade, e.g., clothing, bags, and bottles, in addition to being intentionally manufactured, like those used in industrial abrasives. Microparticles can therefore comprise a large portion of the mass budget of open waters [12]. They can enter waterways through multiple pathways, including wastewater discharge, runoff and wind transport of inadequately disposed waste, littering, and even atmospheric fallout [13], which further complicates efforts to control their abundance as compared with larger debris. Between 4500 and 5200 million metric tons of waste from synthetic polymers are already in the natural environment [14], with global waterways predicted to transport an ever-increasing proportion in coming decades [15].

Lakes are neglected as potential hotspots for the accumulation of anthropogenic debris. As flowing waters like streams and rivers—the primary conduit for moving anthropogenic debris from land into the oceans [16]—enter the still waters of lakes, microparticles will be retained for longer and so may accumulate in higher concentrations. Lakes within fluvial networks can also receive more microparticles overall than coastal areas because they are closer to sources of pollution. Most of the world’s lakes are located within developed northern countries [17], which generate large amounts of solid waste [18,19]. Half of all people in these countries also live within 3 km of freshwater as compared with lower populations immediately around coastlines [20]. The most common types of microparticles, e.g., polypropylene and polyethylene found in bottles and bags [21], may be especially hazardous for lake food webs. Rather than being buried in sediment, these microparticles remain buoyant in the water column because of lower densities than freshwater and so are easily accessible to organisms. Given their proximity to sources of waste, microparticle inputs to lakes are also likely to be younger and less degraded than those in the oceans, resulting in greater potential food web assimilation [22,23].

Although methods to identify and quantify concentrations of anthropogenic microparticles have rapidly advanced in the last decade, an outstanding question is how their abundance relates to human activities and transport routes from land into water [1,2]. Hydrological connectivity in watersheds with human activities should be a major predictor of whether microparticles enter freshwaters. For example, microparticles are generally more abundant in rivers that drain larger catchments with higher population density and more impervious surfaces (e.g., from urbanisation), particularly after rainfall and flooding events [16,24–26]. These catchments can also host large wastewater treatment works (WwTWs), in addition to generating more solid waste that breaks down into secondary particles and films [16]. Textile fibres released by clothes washing, and other primary microparticles (e.g., microbeads), are primarily sourced from the effluent of WwTW [27] and can accumulate in large numbers even after wastewater treatment simply because of the sheer volumes of water that are processed [28]. However, empirical studies typically test the influence of catchment characteristics on microparticle concentrations in a relatively local region and cannot be compared easily because they used different methodologies (e.g., [24,25,29–32]). Understanding why microparticles are more abundant in some places than others requires standardised sampling across large environmental gradients, such as in land use, topography, and hydrology.

Once exported into lakes, in situ water quality can further influence concentrations of anthropogenic microparticles. For example, microparticles can be removed by photodissolution in clear surface waters that attenuate little ultraviolet radiation [33]. Lakes with high levels of microbial activity may similarly remove more microparticles from surface waters. Removal can arise through biofouling that causes particles to sink [34,35] and aggregation with organic detritus in the surface layer [36]. Emerging evidence also suggests that microbes can directly assimilate and mineralise some microparticles [8], such as polyethylene terephthalate and polyamide, through enzymatic cleavage [37,38]. These effects may themselves vary among lakes because of differences in the composition and biomass of resident microbial communities [39].

Here, we surveyed concentrations of anthropogenic microparticles in 67 European lakes distributed across 30° of latitude and spanning large environmental gradients. We related microparticle concentrations in horizontal net tows of surface waters to surrounding land use and water chemistry recorded during our surveys. At a landscape scale, we predicted that microparticle concentrations would be greatest in catchments with more human activity, such as with a larger proportion of urban areas and WwTWs that process large volumes of wastewater [24,25,40]. By contrast, we predicted lower concentrations in catchments with intact natural vegetation, such as forests [24], which can be less impacted by human activity. We also predicted that microparticles concentrations would increase with littering and inadequate waste disposal, which we estimated in surrounding catchments with an existing spatially explicit model of floating plastic emissions in receiving waters [16]. Within lakes, we predicted that the biological and photochemical degradation potential of surface waters would be associated with lower microparticle concentrations, such as from greater microbial activity or deeper penetration of ultraviolet radiation [41].

## Results

### Characterising microplastics from European lakes in a global context

**Fig 1:**
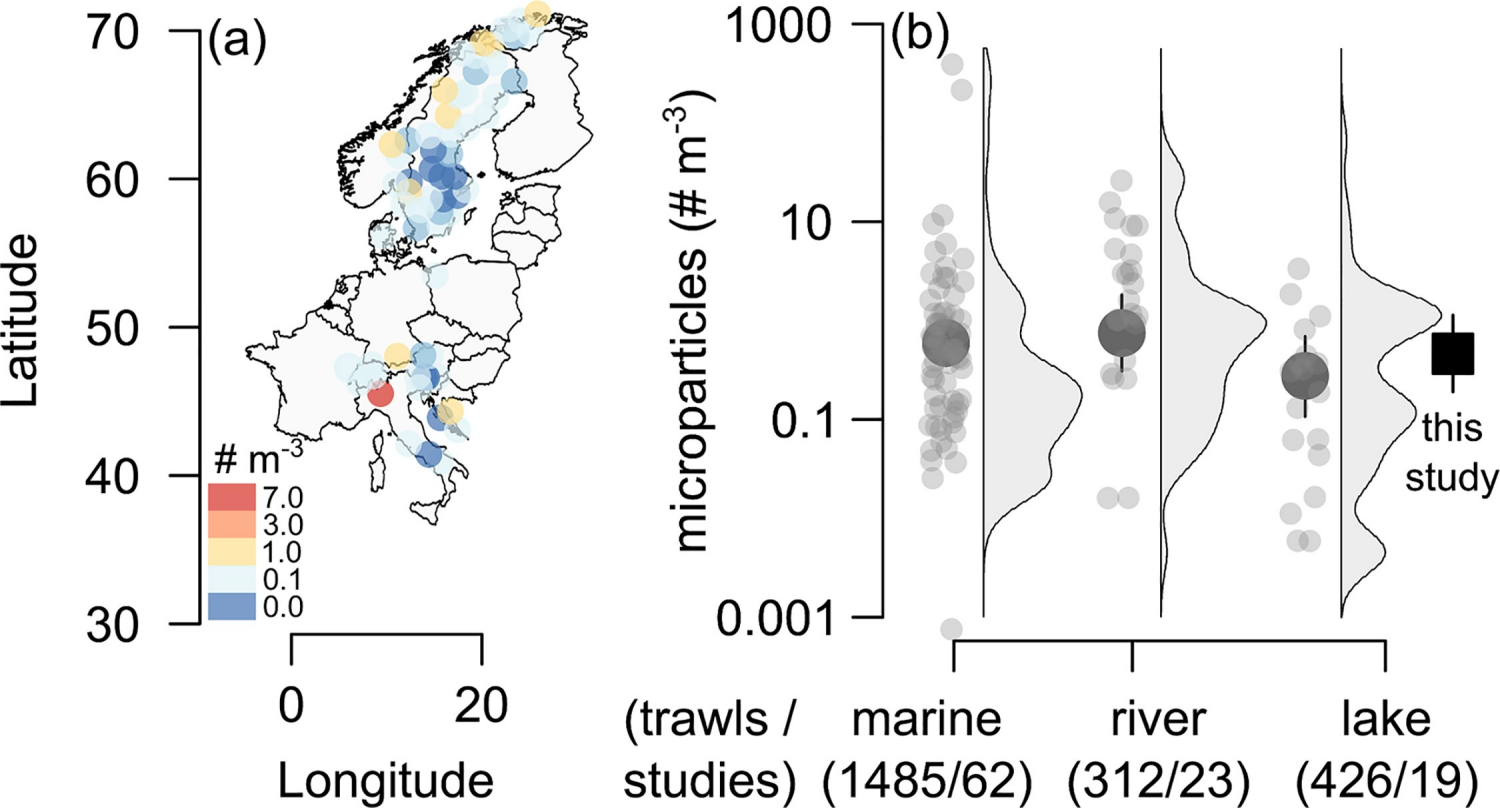
Lakes in Europe harbour more anthropogenic microparticles than elsewhere. **(a)** Concentrations of microparticles (310 to 5,000 μm) across 67 lakes sampled in 2019. **(b)** Microparticle concentrations in surface waters of European lakes from (a) (square symbol) exceeded those estimated, on average, in published studies of lakes, but was similar to marine/estuarine and river environments. Filled circles are estimated means ± 95% CIs compiled from published studies for each environment with square denoting concentrations observed in this study. Numbers beneath habitat types are total number of trawls/unique studies, and values (67/1) from this study are included in lake counts. Studies generally counted particles up to 5,000 μm in size, but some did not specify an upper limit, and lower limits varied from 45 to 780 μm and were accounted for when estimating means in our statistical analysis (see Methods). Published studies were compiled by reviews in [6] (*N =* 55), [47] (*N* = 7), [48] (*N* = 8), [49] (*N* = 2), [50] (*N* = 1), and ourselves (*N* = 23). The data underlying (a) are given in S1 Data, with the base map obtained from Natural Earth (http://www.naturalearthdata.com). The individual points shown in (b) are derived by fitting the model given in S3 Data to raw data in S1 Data, with values of summary statistics given in S2 Table.

Microparticle concentrations in the surface waters of European lakes in our field survey varied considerably between 0 and 7.3 (median = 0.28) particles m^−3^. However, there was no clear latitudinal trend (Spearman correlation test, *ρ* = 0.08, *p* = 0.546; Fig 1A). The overwhelming majority of particles were anthropogenic fibres (92%) either from synthetic or natural sources, consistent with studies of surface freshwaters impacted by human activities (e.g., [42–44]). In a subset of 56 microparticles measured with Fourier transform infrared (FTIR) spectroscopy, 77% were classed as anthropogenic based on vibrational bands, including fibres, particles, and films. The remaining 23% of microparticles of unknown origin were all structurally identified as cellulose fibres. As some natural cellulosic fibres are intentionally produced, such as cotton used for textiles, they cannot be definitively differentiated from other cellulose-based fibres produced in the natural environment such as by algae and plants without reference databases [45,46]. Matching spectra to those in existing databases of anthropogenic and natural materials supported these classifications, except for classifying 3 unknown cellulosic fibres as being sourced from the natural environment (i.e., grass fibres) and another 2 as being anthropogenic. Combining the manual inspection of known vibrational bands with the library matching, 94% of identifiable microparticles came from anthropogenic sources (S1 Table).

To contextualise the microparticle concentrations observed in our field survey, we synthesised existing data from horizontal trawls of lakes, rivers, and marine/estuarine environments. Consequently, we generated the largest and most reproducible, open access database of microparticle concentrations in global surface waters that is currently available, consisting of >2,100 individual net tows (S1 Data). Some studies in our database may have inadvertently classified regenerated or processed natural materials (e.g., synthetic cellulose or textile fibres) as microplastics, but this information cannot be deduced retrospectively [51,52]. Instead, we considered anthropogenic microparticles more broadly and did not differentiate their composition. We then compared the synthesised data with our observed data by fitting a generalised linear mixed model to the net tow concentrations accounting for differences in environment, mesh size and detection limit among studies, in addition to random variation in the studies themselves.

Although we found that lakes in the synthetic database had similar microparticle concentrations to other environments, they were relatively understudied and biased towards sites that were less influenced by anthropogenic pollution (Fig 1B). The 95% confidence interval (CI) for the difference in mean microparticle concentrations between lakes (both from published studies and our field survey) and marine/estuarine environments overlapped zero when accounting for mesh size and detection limit of studies, and lakes had slightly lower concentrations than rivers (95% CIs: −0.08 to 0.71 and 0.06 to 1.25 particles m^−3^, respectively). This result could arise because most lakes from the literature studies were exceptionally large and their microplastic concentrations were negatively correlated with lake area (*ρ* = −0.26, *p* < 0.001, *n =* 359). Of the 18 unique lake studies in our synthetic database, 11 were from sites with a surface area >1,000 km^2^, despite only 0.0002% of lakes worldwide being this size [17]. In the smaller European lakes from our field survey (median area = 1.84 km^2^; range = 0.05 to 1,083 km^2^), where there may be less dilution of anthropogenic debris, microparticle concentrations were higher and no longer differed from river studies (95% CI for difference: −0.20 to 1.21 particles m^−3^; Fig 1B). One explanation for the difference in concentrations between published studies and our field survey is that we considered all microparticles, whereas others may have focused only on microplastics. Microparticle concentrations in lakes generally (i.e., across both published studies and our field survey) may have also not statistically differed from marine/estuarine environments because of their much greater variability (Fig 1B). Lakes can have large regional variation in microparticle concentrations reflecting different sources of pollution. For example, some sites may be dominated by wastewater effluent, urban development, and landfill proximity [24,25,28,29], whereas remote sites may instead obtain most of their particles from airborne transport and deposition [53].

Geographic biases may have also underestimated potential microparticle concentrations in lakes as compared with river studies. Most (82%) of the lake observations in our synthetic database came from affluent nations (e.g., per capita gross domestic product (GDP) >$10,000 2016 USD) with better waste management systems [18,54]. Our European field survey similarly sampled mostly wealthier Northern and Western European countries, with only 5 lakes in former Eastern Bloc countries. Historical disparities in waste management facilities in the latter, particularly in rural areas, may therefore result in higher plastic concentrations than reported here [55]. However, most of the world’s lake area is located at northern latitudes in affluent nations [17], so our results are highly generalizable. These countries can also produce larger absolute quantities of waste per capita, even if a smaller proportion is mismanaged [18].

### Microparticle concentrations vary with land use, hydrology, and in-lake microbial activity

**Fig 2:**
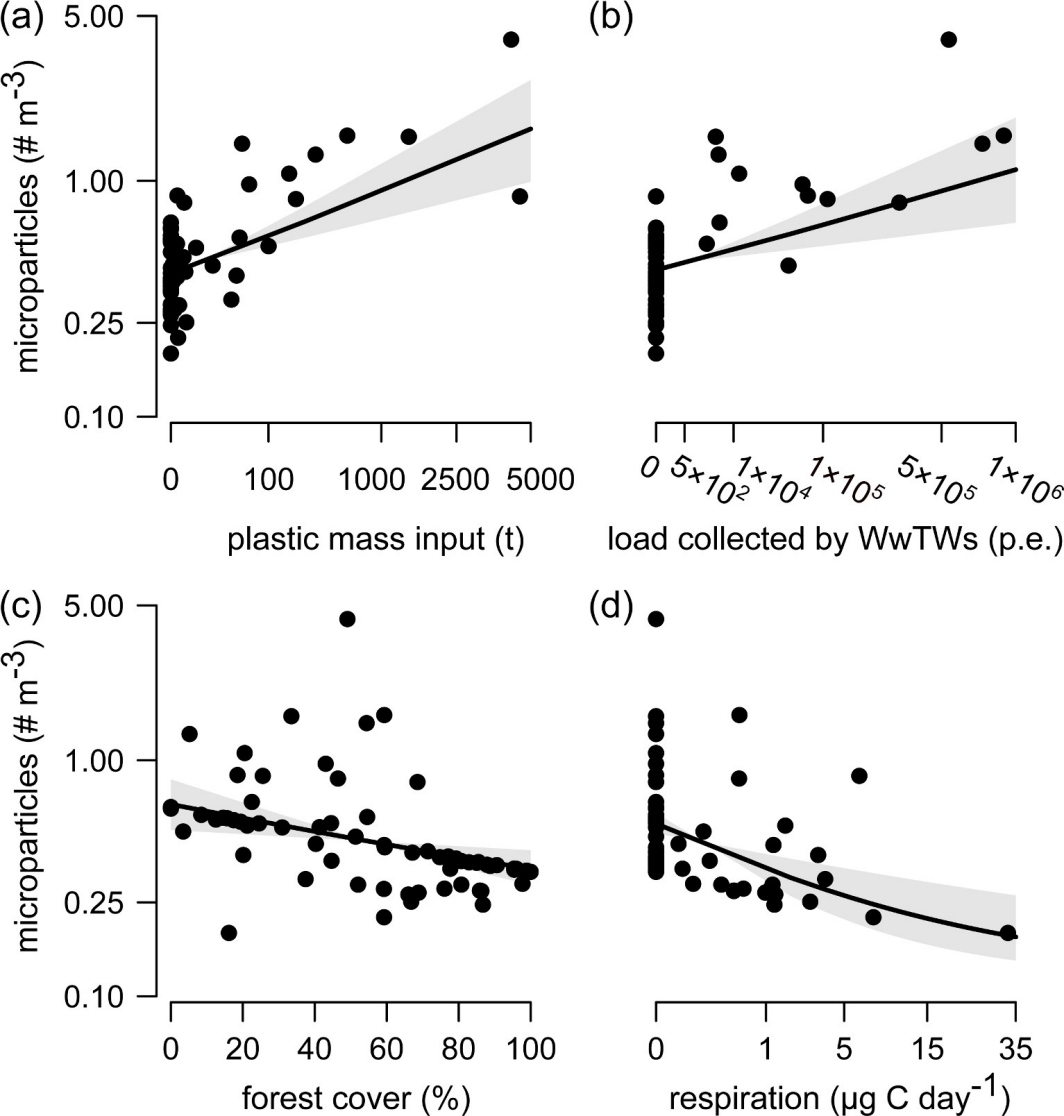
Microparticles are rarer in waters of less disturbed catchments and with more potential for microbial decomposition. Lines show mean model fit (±95% CI) at mean of all other variables for effects of **(a)** estimated plastic mass inputs integrated over the retention time of the lake prior to sampling, **(b)** load of wastewater collected by treatment plants in the surrounding catchment in p.e., **(c)** forest cover in the surrounding catchment, and **(d)** respiration measured in 24-hour laboratory incubations of lake water as an index of organic matter degradation [56]. Mean (95% CI) Bayesian R^2^ = 0.37 (0.21 to 0.54). *N =* 67. Individual data points were derived by fitting the model given in S3 Data to raw data in S2 Data, with values used to generate lines and errors given in S2 Table. p.e., population equivalent; WwTWs, wastewater treatment works.

Human activities were the strongest predictors of surface water microparticle concentrations in the European lakes from our field survey. Concentrations were predicted to quadruple, on average, from 0.47 to 1.92 microparticles m^−3^ over the range of estimated plastic mass inputs (95% CI for effect on log-scale: 0.18 to 0.40; Fig 2A), which integrated population density, size, and runoff generation of each catchment with the retention time of respective lake water. WwTWs had a similarly large positive effect on microparticle concentrations (95% CI: 0.10 to 0.35; Fig 2B). Whether people were in urban environments made little difference (95% CI for effect of urban land use: −0.15 to 0.13), suggesting that their presence alone was enough to generate mismanaged plastic waste (MPW). Likewise, we found more microparticles in catchments with lower forest cover (95% CI: −0.34 to −0.07). Concentrations doubled, on average, from 0.39 to 0.78 particles m^−3^ as surrounding forest cover decreased from 100% to 0% at the mean values of all other variables (Fig 2C).

Microparticle concentrations also varied with the potential for biological but not chemical degradation across lakes. We found fewer microparticles in lakes from our field survey where resident microbial communities degraded organic matter more quickly (95% CI for effect: −0.50 to −0.14), as indicated by higher oxygen consumption rates in short-term laboratory incubations of lake water [56]. Microparticle concentrations decreased by 4.8 times, on average, from 0.64 to 0.13 particles m^−3^ over the range of observed respiration rates at the mean values of all other variables (Fig 2D). This decline was not an artifact of respiration being lower in less disturbed lakes that also received fewer nutrient inputs. Primary productivity as an indicator of trophic state was not correlated with potential respiration (*ρ* = 0.10, *p* = 0.394). The importance of biological degradation can explain why some lakes, even at northern latitudes, had high microparticle concentrations (Fig 1A). Although these sites may be relatively isolated from land use impacts, they will be less likely to remove any microparticles they do receive from local sources, e.g., fishing activities, or atmospheric deposition. More generally, these results suggest that lower in-lake processing may offset reductions in microparticle concentrations from reduced delivery. We also found no association between microparticle concentrations and in-lake photodegradation (95% CI: −0.12 to 0.14), as indicated by the change in UV absorption of lake water with increasing wavelength [57].

## Discussion

Our continent-scale field survey goes beyond describing patterns of anthropogenic microparticles in nature to develop a predictive understanding of the landscape features associated with their abundances. Despite being developed and calibrated for the outflow of major rivers into the world’s oceans, we independently validated a global model of plastic waste inputs to fluvial networks [16]. We found that this model can be used to predict surface microparticles in an entirely new habitat (i.e., lake surface waters), where microparticle accumulation is relatively large but, to date, has been understudied [2]. Moreover, this model of plastic waste emissions was a better predictor of microparticle concentrations in lakes than traditional correlates, e.g., human land use and light penetration [25,41]. Predictions from this model may only improve with future refinements, like considering transport roughness of individual watersheds given their slope and land use, e.g., landfill locations and spatial orientation [58]. Our findings also suggest that the location and load of WwTWs can help predict downstream microparticle concentrations across a large spatial scale, as expected even if they release a relatively small proportion of waste into the environment [28]. Importantly, our results demonstrate the potential of spatially explicit catchment runoff and land use models to help prioritise lakes for pollution mitigation and remediation.

The importance of microbial but not photochemical degradation potential within each lake suggests that some naturally occurring taxa and enzymatic processes may help remove anthropogenic microparticles from the environment. For example, enzymes involved in the hydrolysis of polyethylene terephthalate may be ubiquitous in marine and terrestrial environments [59]. However, not all microparticles are equally amenable to microbial degradation. Polymers vary in their backbone structure. For example, condensation polymers polyethylene terephthalate and polyurethane that contain ester and urethane bonds, respectively, in their main chains may be easier for microbes to oxidise, as compared with addition polymers that are dominated by C–C backbones with few functional groups, such as polypropylene and polyethylene [38,60]. Studies that associate microbial taxa and functions with different plastic types should now be prioritised to help identify candidates for future remediation efforts and inform more biodegradable polymer designs. Higher respiration rates may have also been associated with lower microparticle concentrations because they reflected lakes with more productive communities overall. For example, microparticles can be directly ingested by metazoans and exported from surface waters aggregation with organic detritus [36,61]. Algal biofilms can also colonise floating materials and cause them to degrade and sink, partly by recruiting novel bacterial communities [62].

Our field survey had at least five limitations in quantifying anthropogenic debris of European lakes despite clear associations with catchment-level predictors. First, we excluded macroparticles (>5 mm) that can be generated at high quantities relative to microparticles, including in high-income countries [54,63] and pose a major environmental problem for larger organisms [2]. However, in the case of synthetic materials, macro- and microplastics concentrations tend to be correlated (e.g., [64]), so our sampling may reflect freshwater pollution more broadly. Second, we never sampled ultrapure water through our field equipment as an additional negative control despite procedural blanks in the lab. As we recorded no microparticles in 11 lakes distributed relatively evenly throughout our sampling period, we are confident that our equipment was not a major source of contamination. Third, we did not separate particles with an organic digest or density separation prior to enumeration. Digestion is commonly used to remove nonplastic organic material, especially in samples with high organic content like sediments rather than the surface waters we studied [65]. Our samples were also dominated by fibres, which can be anthropogenic in origin from natural materials, e.g., cotton or wool. Digestion can therefore be undesirable, as it can degrade both naturally derived and synthetic microparticles, especially environmental samples with reduced structural integrity [65,66]. Our use of FTIR instead explicitly accounts for potential contamination by organic particles from the natural environment, and this estimate was relatively low, i.e., 6% of identified microparticles. Fourth, we assumed that we sampled equal volumes across sites. Lakes can, however, vary in surface flow during sampling, but by simulating this source of error in our statistical models, we found that it was unlikely to bias our results. Sample volumes would have had to have varied by ≥20% among sites and be systematically and tightly correlated (i.e., *r* > 0.6) with observed microparticle concentrations so that the effects of catchment characteristics were no longer statistically significant (S1 Fig). Differences in vertical mixing among lakes may similarly have influenced the observed microparticle concentrations [67] but again would have had to have systematically diluted sampling volumes (*r* > 0.6) among sites to bias our statistical models (S1 Fig). Prevailing wind conditions could instead account for some of the variation remaining unexplained in our statistical models [67], though may be less important for fibres [68]. Finally, local waste sources can also contribute to variation in our observed concentrations and may have not been entirely captured by our model predictors. For example, our model of MPW emissions assumes that per capita solid waste generation scales with GDP within countries [18]. This assumption is weakly supported across our study region (S2 Fig) and may be more variable elsewhere. Importantly, our work provides a valuable test of the local and landscape features that predict microparticle concentrations across European lakes even if some variation remains unexplained.

## Conclusions

As anthropogenic debris from both synthetic and natural sources will continue to pollute the environment for decades [14,15]—even with improved waste management and mitigation [18,54]—interventions need to identify the most impacted sites. Our study advances this challenge in two major ways. First, using the single largest field survey of lakes to date, we show that both catchment-specific estimates of waste generation and water quality can accurately predict the microparticle concentrations of surface waters (S2 Table). The former predictor, specifically the estimated plastic mass input to lakes, also incorporates temporal variation in the delivery potential of debris from surrounding catchments [16], so it can be used to generate predictions at intra-annual timescales. Our survey also provides a valuable baseline for future comparisons. Despite concerns that net trawls underestimate microparticle concentrations, such as because of large mesh sizes used to avoid clogging over large distances, filtering smaller grab samples (e.g., <20 L) through finer meshes is also problematic. The latter approach has high variability, such as from local water patterns, making it difficult to extrapolate spatially [69]. Second, our synthetic net trawl database will enable consistent intercalibration among studies of anthropogenic debris to contextualise local observations. Most microparticle studies liken their observations to the literature, but often do so ad hoc with no consistent comparison. Our analysis of this new database also shows that differences in mesh sizes can be corrected statistically (S2 Table), at least over our range of observed values: 45 to 780 μm. Together, our study provides a valuable evidence base to help prioritise monitoring and mitigation of anthropogenic debris in the world’s lakes.

## Methods

### Study sites

Between April and September 2019, we sampled 67 lakes and delineated the land that drained into each of them, hereafter “catchment”. Lakes were selected for a larger survey based on accessibility and having dissolved organic carbon concentrations <15 mg L^−1^. They varied in size from 0.05 to 1,083 (median = 1.84) km^2^, with a mean depth of 2.3 to 119.8 (median = 8.0) m [70]. Catchments were delineated by mapping flow direction and accumulation from the 25 m European Digital Elevation Model (EU-DEM) v1.1 [71]. First, landscape flow direction was estimated using the D8 algorithm and accumulation grids for the EU-DEM with the “Breach Depression” algorithm from the whitebox v1.0.2 package [72] in R v.3.6. Using the “Watershed” algorithm, we then identified which pixels contributed runoff to each lake based on the flow direction grid. The highest flow accumulation value within each lake polygon, analogous to the outflow, was selected as the pour point. Consequently, all land that drained into the pour point was classified as the lake catchment. When the area surrounding the outflow was particularly flat, the flow direction algorithm often underestimated the surrounding contributing area. Thus, we added a 25-m buffer (i.e., 1 pixel) to each lake polygon in these cases to reduce underestimation of catchment area. We then summarised surrounding land cover by clipping the 100-m resolution CORINE Land Cover 2018 image produced by the European Environment Agency (EEA) [73] to each catchment. We extracted forest and urban land cover as the proportion of pixels in each catchment that were either in broadleaved, coniferous, or mixed forest or continuous and discontinuous urban fabric, respectively. Finally, we extracted the load of wastewater collected by all the treatment plants in each catchment during 2018 by clipping the EEA’s Urban Waste Water Treatment Directive (UWWTD) database [74] to each catchment. Values were expressed in population equivalent, defined by the UWWTD of the European Union (91/271/EEC) to represent the organic biodegradable load equivalent to a 5-day biochemical oxygen demand of 60 g day^−1^.

### Estimates of mismanaged plastic waste emissions

For each catchment, we estimated plastic mass inputs to each lake from monthly averages of catchment runoff and MPW generated on surrounding lands by littering and inadequate disposal practices. We first extracted daily MPW generation in tonnes per km^2^ from a global 30 × 30 arc-seconds (ca. 1 km) resolution grid estimated from population density (30 × 30 arc-seconds) and country-scale waste generation and management statistics [18]. As levels of plastic consumption and waste management infrastructure vary at local to regional scales, we corrected MPW generation with gridded subnational estimates of GDP per capita as described in [18]. Plastic mass emitted into each lake from the surrounding catchment was then estimated on a monthly interval from MPW generated on surrounding lands and runoff using the empirical formula in [16]. Average monthly runoff expressed in mm per day was sourced from an observation-based global gridded dataset at a 0.5-degree (ca. 55 km) spatial resolution [75]. We then summed plastic mass emissions over the water residence time of each lake extracted from HydroLAKES v1.0 [70].

### Field sampling

We performed a single trawl for 100 m in a random direction starting from the deepest point of each lake. The deepest point is commonly used in water quality surveys because it is considered most representative of the entire catchment and minimises internal mixing processes [76]. Distance was mapped with a STRIKER Plus 4cv GPS fishfinder (Garmin International, USA). There was always 100 m of water for sampling, though the small sizes of some lakes meant that we occasionally reached into littoral zones. For this reason, transects could not be longer if we wanted to employ a consistent methodology, and the sizes of lakes generally meant that our sampling transect covered a much higher proportion of surface area than in marine surveys. We also collected a surface grab sample from the deepest point of each lake to measure potential biological and photochemical decomposition.

Trawls were performed with a 300-mm diameter plankton net (880-mm long bag with 250-μm nylon mesh). The net was connected to a 50-mm screw-on filter with 310-μm stainless steel mesh and submerged just beneath the surface outside of the wake of the boat. After each trawl, steel meshes were wrapped in precombusted (450°C, 4 hours) aluminium foil in the field and stored until analysed in the lab. By trawling surface waters, we may have biased our sampling towards higher density microparticles that remain buoyant in the water column. However, this approach specifically allowed us to test our focal prediction that microparticles were removed from surface waters by high levels of biological and photochemical degradation. We were confident that our net shed no fibres, as we recorded no microparticles in 11 lakes distributed throughout the entire sampling period rather than exclusively later in sampling when the net might have been worn.

We calculated sample volumes as 7,100 L by multiplying the area of the submerged net mouth by the distance the net was towed after [47]. We assumed that currents in the lakes were negligible during sampling, especially relative to boat speed, and so sample volumes would not vary among sites. While we recognise this volume is small for marine studies, it is within the range commonly reported in freshwaters (reviewed by [47]) and far exceeds the recommended minimum sample volume of 500 L [49].

### Decomposition assays

In the lab, we incubated 20 mL of unfiltered lake water in duplicate glass bottles with no headspace in the dark for 24 hours at room temperatures. Bottles were sealed gas tight with rubber lids and a crimp top. Two additional bottles received ca. 34,000 lux from a cold 5,000K lamp for 3 hours. We recorded dissolved O_2_ concentrations at the start and end of the incubation in each bottle with an optical sensor (OXY-1 ST, PreSens, Germany). Total respiration and primary productivity rates were calculated as the difference between O_2_ concentration at the start and end of the incubation averaged across dark and light bottles, respectively. We assumed a respiratory quotient of one [56], though this made no difference to our results as it was a constant among lakes and our comparisons were focused on lakes in our study only. Although we also measured total respiration, filtering our samples to only the microbial fraction would have removed all particle-attached microbes, which are arguably the most active in decomposition [77]. The lack of filtering was further supported by the lack of any visible zooplankton in the incubations, and correcting our measurements to only bacterial respiration using primary productivity rates as in [78] made no difference to our results (S2 Table). In the lab, we also measured the optical properties of 4 mL of water that had been passed through a precombusted (450°C, 4 hours) glass fibre filter (0.5-μm nominal pore size, Macherey-Nagel, Germany). We measured ultraviolet-visible absorption between 200 and 700 nm in a 1-cm path length quartz cuvette using a portable spectrophotometer (FLAME-DA-CUV-UV-VIS with Flame-S-UV-VIS, Ocean Optics, USA). We then calculated the single exponential decay from 275 to 295 nm using a linear fit between log-transformed absorbance values and wavelength [57].

### Microparticle identification

In the lab, we first picked all visible particles from the steel mesh with steel tweezers into a glass plate. The steel mesh was then rinsed from behind into the glass plate with ca. 10 mL of deionised water from a polypropylene syringe. We also rinsed the aluminium foil that stored the mesh postsampling. Glass plates and steel tools were rinsed with deionised water and inspected between samples beneath a stereomicroscope. Glass plates were kept closed as much as possible.

All microparticles were visually counted and classified under a GXM-XTL stereomicroscope (GT Vision, Suffolk, UK) at up to 40 times magnification as fibres, clear or coloured particles, or films [79]. We excluded particles with visible cellular or organic structures. We also differentiated between whether fibres were anthropogenic (either plastic or nonplastic origin) or naturally derived from animal or plant material in the environment based on visual observation of morphology, structure, and physical response under a stereomicroscope, following procedures outlined by [45,80]. Specifically, fibres that were lustrous, uneven in thickness along their length, heterogeneously coloured, or appeared twisted with ribbon-like folds were excluded. Fibres of unknown origin were further tested for their tensile strength with tweezers, excluding those that broke with little force. Once analysed, the mesh disks were wrapped in aluminium foil, and the liquid with all particles returned to their original container. Later, 20% of samples were randomly selected and recounted blind to the original data, always with the same result. Throughout, we wore cotton laboratory coats and latex gloves and never observed particles from these items in our samples. Most samples were processed in 10 minutes, and all under 20 minutes. Contamination from laboratory air during sample processing was also negligible. As an additional control in our workflow, we exposed open petri dishes lined with filter paper for 2 hours during microscopy work. We recorded a mean (±standard deviation) of 0.02 ± 0.02 particles m^−3^ (all fibres) when counts were divided by our sampling volume over our maximum sampling time, so no corrections were applied.

We further validated particle classifications using attenuated total reflection (ATR) FTIR spectroscopy. Particles were pressed directly on the ZnSe crystal (4-mm^2^ area) of a Thermo Scientific Nicolet iS50 FTIR spectrometer with built-in ATR module after cleaning the crystal with acetone. We collected 32 scans at a resolution of 4 cm^−1^ between 400 to 4,000 cm^−1^ and subtracted a background scan from each sample. We also measured 4 positive controls consisting of a polyamide pellet, polyester fibre, polyethylene powder, and polyvinyl chloride powder. Spectra were denoised with Savitzky–Golay smoothing using a third-order polynomial over a 20-cm^−1^ interval and corrected with a 15% adaptive baseline before normalising peak maxima to 1 using SpectraGryph 1.2.15 [81], as recommended by [82]. Each spectrum was then manually inspected and classified based on known vibrational bands [46,83,84] as either anthropogenic, which included synthetic and modified natural materials, naturally derived from the local environment, such as from algae, vegetation, or animal fur, or unknown. Although automated pipelines exist, manual inspection is considered the gold standard to avoid misidentification [82,84]. Nonetheless, we also performed automated library matching of entire spectra in the region [46,84] between 700 to 1,850 cm^−1^ using Open Specy [85–87]. We accepted matches with the highest Pearson correlation coefficient *r* of at least 0.90; *r* ranged between 0.95 and 0.98 in the controls.

To assess the reliability of our study, we also scored our methods according to widely accepted criteria in the field [49]. Our overall reliability score of 11.00 exceeded the mean of 8.41 (interquartile range: 6 to 10) reported from 56 studies assessed by [49]. This score resulted from reporting reproducible sampling methods (2 points), trawling above recommended amounts for surface waters of 500 L (2 points), reporting that samples were stored in precombusted containers (2 points), employing recommended contamination mitigation in laboratory preparation, e.g., cotton lab coats and wiping surfaces (2 points), mitigating airborne contamination as much as possible and monitoring levels in parallel controls (1 point), using negative controls to monitor lab processing only (1 point), and identification of a subset of polymers using FTIR spectroscopy (1 point).

### Global net trawl database

To contextualise our field survey, we compiled a synthetic database on published microparticle concentrations collected solely using horizontal net tows of surface waters. Studies in our database came from the recent systematic review of [6], which we cross-referenced against microplastic reviews with quantitative estimates to identify additional data sources [2,47–50,79,88,89]. We further supplemented these sources with a Google Scholar search on 7 September 2020 for material published since 2019 with the terms “microplastics,” “trawls,” and “lakes.” We then extracted individual net tow values for each study where possible (*N =* 1,989/2,156 observations). As in [47], we converted concentrations reported per unit area to volumetric units by multiplying the area of the net mouth submerged in the water column by sampling distance.

### Statistical analyses

We first fitted a generalised linear mixed model to the microparticle concentrations in our field survey to test if they differed from other habitats in the synthetic database. Given that microparticles are ubiquitous in all habitats on Earth, any zero concentrations are likely to arise because they are beneath a minimum but unknown detection limit. We therefore treated zeros as partially known (i.e., censored), laying somewhere between a detection limit [90,91]. Concentrations could then be modelled from a censored log-normal distribution with mean varying with habitat type (marine/estuarine, lake, or river) and the mesh size used in sampling. We accounted for additional variation in field sampling and particle identification among studies by sampling study-specific intercepts from a zero-normal distribution with estimated standard deviation.

We next fitted a Poisson hurdle model to test predictors of microparticle counts across our 67 study lakes. This model effectively estimated the probability of detecting microparticles across all our samples by fitting a Bernoulli function to the binary outcome of whether counts were zero (*N =* 11/67) or positive, and then fitting a separate, zero-truncated Poisson distribution to the positive counts. We let the log-transformed mean of the Poisson model vary with a linear combination of the estimated plastic mass inputs to each lake, both forest and urban land cover and total wastewater load collected in the surrounding lake catchment, total respiration from incubations of lake water, and the spectral slope of UV absorption in lake water. Spectral slope was log-transformed to deal with high skewness, whereas we used a cube-root transformation for respiration and plastic mass input due to some zero values.

All models were fitted using Hamiltonian Monte Carlo sampling by calling RStan v2.19 from R v3.6 [92]. We simulated 4 Markov chains for each model and assigned weakly informative normal priors for all parameters [93]. All predictors were standardised to a mean of 0 and SD of 1 to compare the relative importance of their effects. Model convergence was assessed by visually inspecting chain traces and posterior predictive checks [94]. We ensured that the potential scale reduction factor and effective number of samples for the posterior of each parameter were ≤1.01 and ≥800, respectively [93]. We also tested the influence of individual observations with leave-one-out cross-validation using Pareto smoothed importance sampling [95]. Model effects were inferred by calculating 95% credible intervals (CIs) from a subset of 1,000 simulations, and we calculated a Bayesian *R*^2^ to summarise model fit [96]. Data and R code to reproduce these analyses are given in S1 to S4 Data.

## Supporting information

S1 Fig Little bias in model estimates from uneven sampling volumes. To test the influence of sampling volume on our model estimates, we assumed volumes varied with a standard deviation (*σ*) equal to (a) 20% or (b) 40% of the mean *μ* = 7,100 L. We then randomly sampled volumes from a normal distribution with these parameters (i.e. *μ* and *σ*), assuming volumes were correlated with observed microparticle concentrations with a Pearson correlation coefficient *r* = 0.0 to 0.8 in 0.1 intervals. We then refit the model predicting microparticle concentrations in 67 European lakes described in the main text including sampling volume as an additional predictor. We generated 100 replicates for each *σ* and *r* combination. Lines are the probability (*p*) of finding an effect of sampling volume (black line), i.e., proportion of 100 simulations where 95% CIs for model effect excluded zero. We also plotted the probability of no longer having an effect of estimated plastic mass inputs (grey line), total wastewater load (brown line), forest cover (green line), and total respiration (blue line), i.e., proportion of times 95% CIs overlapped zero. Grey shading denotes *p* = 0.95, values above that indicate *σ* and *r* where sampling volume could be considered a statistically significant predictor of microparticle concentrations and other variables could be considered to no longer be statistically significant. As this was a simulation with random sampling, we give the R code to reproduce the analysis in S3 Data rather than the raw data underlying the plotted curves. CI, credible interval.
(PDF)

S2 Fig Per capita waste generation scales with per capita GDP in Europe. Each point is a NUTS 2 level used by the European Union for subdividing countries for statistical purposes and was coloured according to corresponding country (see legend inset). Population ranges between 800,000 and 3,000,000 people in NUTS 2 regions and was the smallest spatial scale at which data were available. We used the most recent year available (2013 for all but Spain and Romania where 2012 data were used) and differentiated countries sampled by our field survey (circles) from elsewhere (squares). We sourced all available data from Eurostat (https://ec.europa.eu/eurostat) and divided municipal waste generation (tonnes) in each NUTS 2 region by the proportion of municipal waste collection and expressed values relative to population size in January of the corresponding year (only 2014 population data were available for 6 regions in Poland). We then fitted a linear mixed effects model using RStan as described in the main text to predict per capita waste generation. The only predictor was GDP per capita expressed as a percentage of the EU27 average in 2020, calculated in purchasing power standards that eliminates differences in price levels among countries. We included country as a random effect to account for repeated measurements within EU member states. Solid line is mean ± 95% CI for model fit, mean *R*^2^ (95% CI) = 0.52 (0.44–0.59). Individual data points are given in S4 Data, with lines and errors generated by fitting model given in S3 Data to raw data in S4 Data. CI, credible interval; GDP, gross domestic product; NUTS, Nomenclature of Territorial Units for Statistics.
(PDF)

S1 Table Microparticle composition in field survey of 67 European lakes. Particles were separated into 4 different morphologies and classified with FTIR spectroscopy as being intentionally manufactured (including natural materials used for textiles), naturally occurring in the local environment (e.g., from algae, plants, animals), or having an unknown origin. Samples were selected for the FTIR survey in similar proportion to their percent composition among all microparticles recorded in the field survey. FTIR, Fourier transform infrared.
(DOCX)

S2 Table Estimated parameters from statistical models. For each model response denoted by italics, we report the mean and 95% CI for estimated parameters. Bolded parameters denote statistically significant effects, i.e., 95% CI not overlapping zero. CI, credible interval.
(DOCX)

S1 Data Surface microparticle trawl database. (TXT)

S2 Data Predictors of microparticle concentrations in European lakes. (TXT)

S3 Data R code to reproduce statistical analysis. (TXT)

S4 Data Per capita waste generation and gross domestic product in Europe. (TXT)

## Abbreviations

ATR: attenuated total reflection
CI: credible interval
EEA: European Environment Agency
EU-DEM: European Digital Elevation Model
FTIR: Fourier transform infrared
GDP: gross domestic product
MPW: mismanaged plastic waste
UWWTD: Urban Waste Water Treatment Directive
WwTWs: wastewater treatment works
