# Supplementary figures and images for: Microplastics and anthropogenic fibre concentrations in lakes reflect surrounding land use
Source: PLoS Biol. 2021 Sep 14;19(9):e3001389. doi: 10.1371/journal.pbio.3001389 (PMC8439457; doi:10.1371/journal.pbio.3001389)

(a)  $\sigma = 0.2\mu$

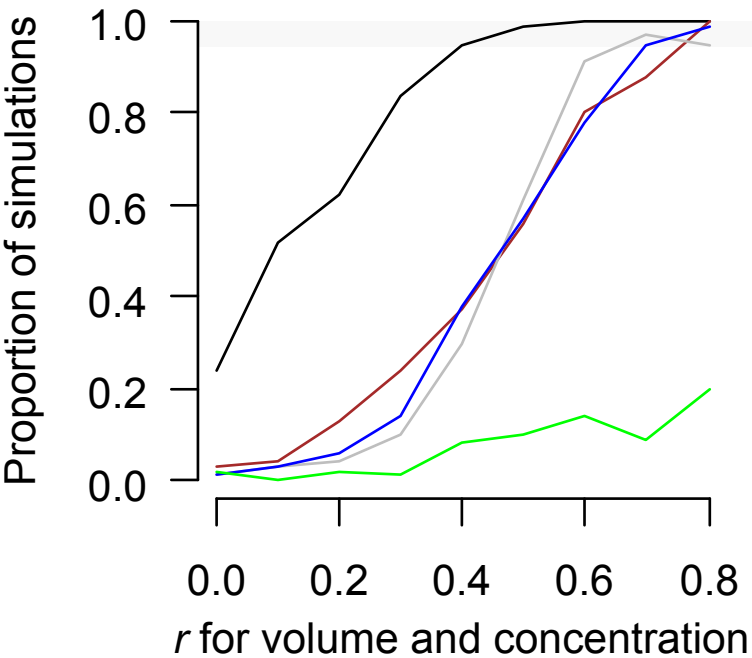

(b)  $\sigma = 0.4\mu$

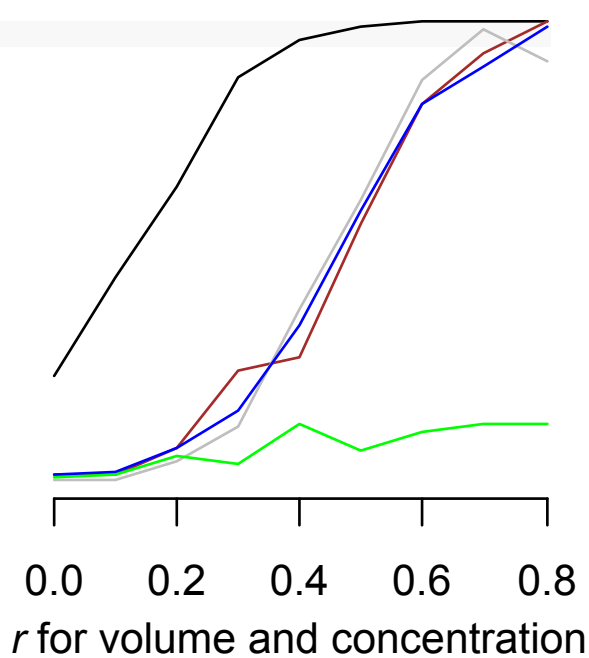

Supplement: S1 Fig — To test the influence of sampling volume on our model estimates, we assumed volumes varied with a standard deviation (σ) equal to (a) 20% or (b) 40% of the mean μ = 7,100 L. We then randomly sampled volumes from a normal distribution with these parameters (i.e. μ and σ), assuming volumes were correlated with observed microparticle concentrations with a Pearson correlation coefficient r = 0.0 to 0.8 in 0.1 intervals. We then refit the model predicting microparticle concentrations in 67 European lakes described in the main text including sampling volume as an additional predictor. We generated 100 replicates for each σ and r combination. Lines are the probability (p) of finding an effect of sampling volume (black line), i.e., proportion of 100 simulations where 95% CIs for model effect excluded zero. We also plotted the probability of no longer having an effect of estimated plastic mass inputs (grey line), total wastewater load (brown line), forest cover (green line), and total respiration (blue line), i.e., proportion of times 95% CIs overlapped zero. Grey shading denotes p = 0.95, values above that indicate σ and r where sampling volume could be considered a statistically significant predictor of microparticle concentrations and other variables could be considered to no longer be statistically significant. As this was a simulation with random sampling, we give the R code to reproduce the analysis in S3 Data rather than the raw data underlying the plotted curves. CI, credible interval. (PDF) [file pbio.3001389.s001.pdf]

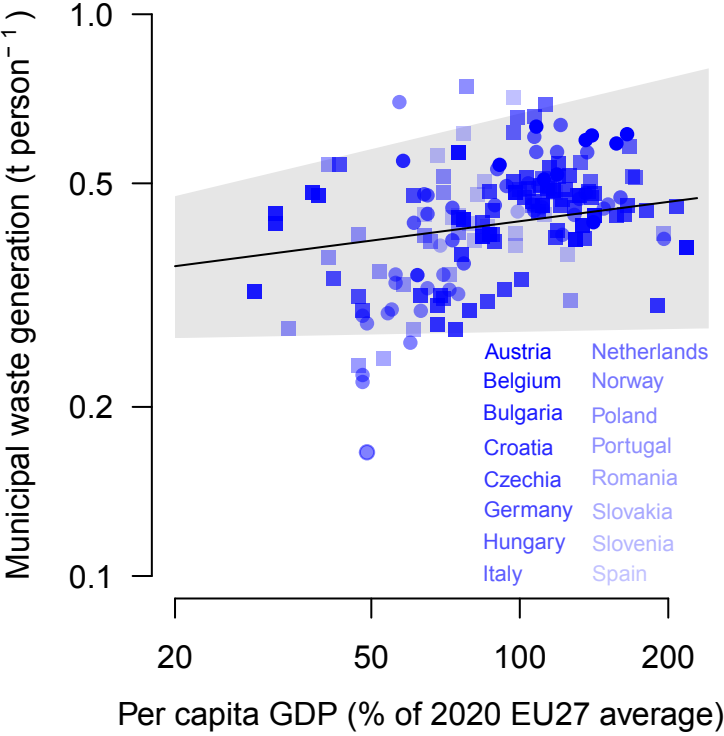

Supplement: S2 Fig — Each point is a NUTS 2 level used by the European Union for subdividing countries for statistical purposes and was coloured according to corresponding country (see legend inset). Population ranges between 800,000 and 3,000,000 people in NUTS 2 regions and was the smallest spatial scale at which data were available. We used the most recent year available (2013 for all but Spain and Romania where 2012 data were used) and differentiated countries sampled by our field survey (circles) from elsewhere (squares). We sourced all available data from Eurostat (https://ec.europa.eu/eurostat) and divided municipal waste generation (tonnes) in each NUTS 2 region by the proportion of municipal waste collection and expressed values relative to population size in January of the corresponding year (only 2014 population data were available for 6 regions in Poland). We then fitted a linear mixed effects model using RStan as described in the main text to predict per capita waste generation. The only predictor was GDP per capita expressed as a percentage of the EU27 average in 2020, calculated in purchasing power standards that eliminates differences in price levels among countries. We included country as a random effect to account for repeated measurements within EU member states. Solid line is mean ± 95% CI for model fit, mean R2 (95% CI) = 0.52 (0.44–0.59). Individual data points are given in S4 Data, with lines and errors generated by fitting model given in S3 Data to raw data in S4 Data. CI, credible interval; GDP, gross domestic product; NUTS, Nomenclature of Territorial Units for Statistics. (PDF) [file pbio.3001389.s002.pdf]
