# Supplementary material for: Microplastics and anthropogenic fibre concentrations in lakes reflect surrounding land use
Source: PLoS Biol. 2021 Sep 14;19(9):e3001389. doi: 10.1371/journal.pbio.3001389 (PMC8439457; doi:10.1371/journal.pbio.3001389)
Supplement: S1 Table — Particles were separated into 4 different morphologies and classified with FTIR spectroscopy as being intentionally manufactured (including natural materials used for textiles), naturally occurring in the local environment (e.g., from algae, plants, animals), or having an unknown origin. Samples were selected for the FTIR survey in similar proportion to their percent composition among all microparticles recorded in the field survey. FTIR, Fourier transform infrared. (DOCX) [file pbio.3001389.s003.docx]

**Supporting Information for Microplastics and anthropogenic fibre concentrations in lakes reflects surrounding land use**

Andrew J. Tanentzap, Samuel Cottingham, Jérémy Fonvielle, Isobel Riley, Lucy M. Walker, Samuel. G. Woodman, Danai Kontou, Christian M. Pichler, Erwin Reisner, Laurent Lebreton

**S1 Table. Microparticle composition in field survey of 67 European lakes**. Particles were separated into four different morphologies and classified with FTIR spectroscopy as being intentionally manufactured (including natural materials used for textiles), naturally occurring in the local environment (e.g. from algae, plants, animals), or having an unknown origin. Samples were selected for the FTIR survey in similar proportion to their percent composition amongst all microparticles recorded in the field survey.

| Morphology | FTIR source classification | | | % in FTIR survey | % in field survey |
| --- | --- | --- | --- | --- | --- |
|  | % anthropogenic | % natural | % unknown |  |  |
| Fibres | 77.6 | 6.1 | 16.3 | 89.3 | 92.3 |
| Clear particles | 100.0 | 0.0 | 0.0 | 3.6 | 1.5 |
| Coloured particles | 100.0 | 0.0 | 0.0 | 5.3 | 4.7 |
| Films | 100.0 | 0.0 | 0.0 | 1.8 | 1.5 |
