# Supplementary material for: Microplastics and anthropogenic fibre concentrations in lakes reflect surrounding land use
Source: PLoS Biol. 2021 Sep 14;19(9):e3001389. doi: 10.1371/journal.pbio.3001389 (PMC8439457; doi:10.1371/journal.pbio.3001389)
Supplement: S2 Table — For each model response denoted by italics, we report the mean and 95% CI for estimated parameters. Bolded parameters denote statistically significant effects, i.e., 95% CI not overlapping zero. CI, credible interval. (DOCX) [file pbio.3001389.s004.docx]

**Supporting Information for Microplastics and anthropogenic fibre concentrations in lakes reflects surrounding land use**

Andrew J. Tanentzap, Samuel Cottingham, Jérémy Fonvielle, Isobel Riley, Lucy M. Walker, Samuel. G. Woodman, Danai Kontou, Christian M. Pichler, Erwin Reisner, Laurent Lebreton

**S2 Table. Estimated parameters from statistical models**. For each model response denoted by italics, we report the mean and 95% credible interval for estimated parameters. Bolded parameters denote statistically significant effects, i.e. 95% CI not overlapping zero.

| Parameter | Mean (95% CI) |
| --- | --- |
| *Net trawl concentrations*, *R^2^ =* 0.68 (0.66 – 0.70) | |
| Mean microparticle concentration (log-scale) | -7.43 (-7.96 – -6.92) |
| Effect of lake habitats | -0.77 (-1.71 – 0.15) |
| Effect of river habitats | 0.23 (-0.66 – 1.12) |
| **Effect of mesh size** | **-1.10 (-1.78 – -0.18)** |
| Variation among studies | 2.07 (1.77 – 2.39) |
| Variation in effect of mesh size among habitats | 0.56 (0.06 – 1.48) |
| Residual variation | 1.44 (1.39 – 1.49) |
| *European lake concentrations* *with total respiration*, *R^2^ =* 0.37 (0.21 – 0.54) | |
| Mean probability of not observing microparticles (logit-scale) | -1.60 (-2.27 – -1.01) |
| Mean microparticle concentration (log-scale) | 1.30 (1.18 – 1.48) |
| **Effect of plastic mass input** | **0.29 (0.18 – 0.40)** |
| **Effect of forest cover** | **-0.21 (-0.34 – -0.07)** |
| Effect of urban land cover | <-0.01 (-0.15 – 0.13) |
| **Effect of wastewater treatment works** | **0.22 (0.10 – 0.35)** |
| **Effect of respiration** | **-0.31 (-0.50 – -0.14)** |
| Effect of UV absorption | 0.02 (-0.12 – 0.14) |
| *European lake concentrations* *with bacterial respiration*, *R^2^ =* 0.37 (0.22 – 0.55) | |
| Mean probability of not observing microparticles (logit-scale) | -1.60 (-2.27 – -0.97) |
| Mean microparticle concentration (log-scale) | 1.33 (1.18 – 1.47) |
| **Effect of plastic mass input** | **0.29 (0.18 – 0.41)** |
| **Effect of forest cover** | **-0.21 (-0.36 – -0.06)** |
| Effect of urban land cover | <0.01 (-0.15 – 0.14) |
| **Effect of wastewater treatment works** | **0.23 (0.09 – 0.34)** |
| **Effect of respiration** | **-0.31 (-0.49 – -0.14)** |
| Effect of UV absorption | 0.02 (-0.12 – 0.15) |
